# Successful occlusion of a bronchobiliary fistula using percutaneous microwave ablation: a case report

**DOI:** 10.3389/fmed.2026.1718950

**Published:** 2026-02-02

**Authors:** Dong Yang, Xiao Zhang, Guijuan Wang, Guang Liu, Jundong Yang

**Affiliations:** 1 Department of Oncology, Affiliated Hospital of Jining Medical University, Jining, Shandong, China; 2 Department of Oncology, Wenshang County People’s Hospital, Jining, Shandong, China; 3 Department of Radiotherapy, Affiliated Hospital of Jining Medical University, Jining, Shandong, China

**Keywords:** bilioptysis, bronchobiliary fistula (BBF), colon cancer, liver metastasis, percutaneous microwave ablation (PMWA)

## Abstract

Bronchobiliary fistula (BBF) is an exceptionally rare and complex clinical entity that involves abnormal communication between the biliary system and the bronchial tree. This article presents the first reported case of BBF successfully occluded with percutaneous microwave ablation (PMWA). A 35-years-old female with primary colon cancer developed BBF after PMWA for liver metastasis and presented with bilioptysis that persisted despite conventional antibiotic therapy and drainage. Under computed tomography (CT) guidance, targeted PMWA with an ablation power of 40 w was applied to the fistula tract for 7 min, resulting in complete radiographic closure and resolution of symptoms. Compared with traditional surgical, endoscopic, or drainage-based approaches, PMWA represents a minimally invasive alternative. While this technique has significant advantages in terms of precision, quick recovery, and applicability to high-risk patients, its efficacy may be limited in patients with large or anatomically complex fistulas. Further clinical validation is needed to establish long-term outcomes and refine technical parameters. This case supports PMWA as a promising therapeutic option for BBF and highlights its potential in fistula management.

## Introduction

Bronchobiliary fistula (BBF) is an extremely rare and challenging pathological condition, that mainly refers to the formation of an abnormal passage between the bronchial tree and the intrahepatic or extrahepatic bile ducts (1). Current therapeutic options for BBF are limited in several key aspects: conservative management and percutaneous transhepatic cholangial drainage (PTCD) rarely achieve definitive fistula closure (2, 3); endoscopic interventions can occlude the fistula via embolization or sealing but are associated with a risk of ectopic embolism (4); and although surgical repair allows direct fistula management, this approach is associated with significant trauma and risk of other complications (5). Therefore, a treatment that enables anatomical closure, offers precise intervention with minimal trauma is clearly needed.

Percutaneous microwave ablation (PMWA) is a commonly used minimally invasive treatment, but its application in the treatment of BBF has not been reported (6–8). This paper reports a case in which BBF was successfully treated with PMWA and aims to provide new ideas and methods for the clinical treatment of this disease.

## Case presentation

**FIGURE 1:**
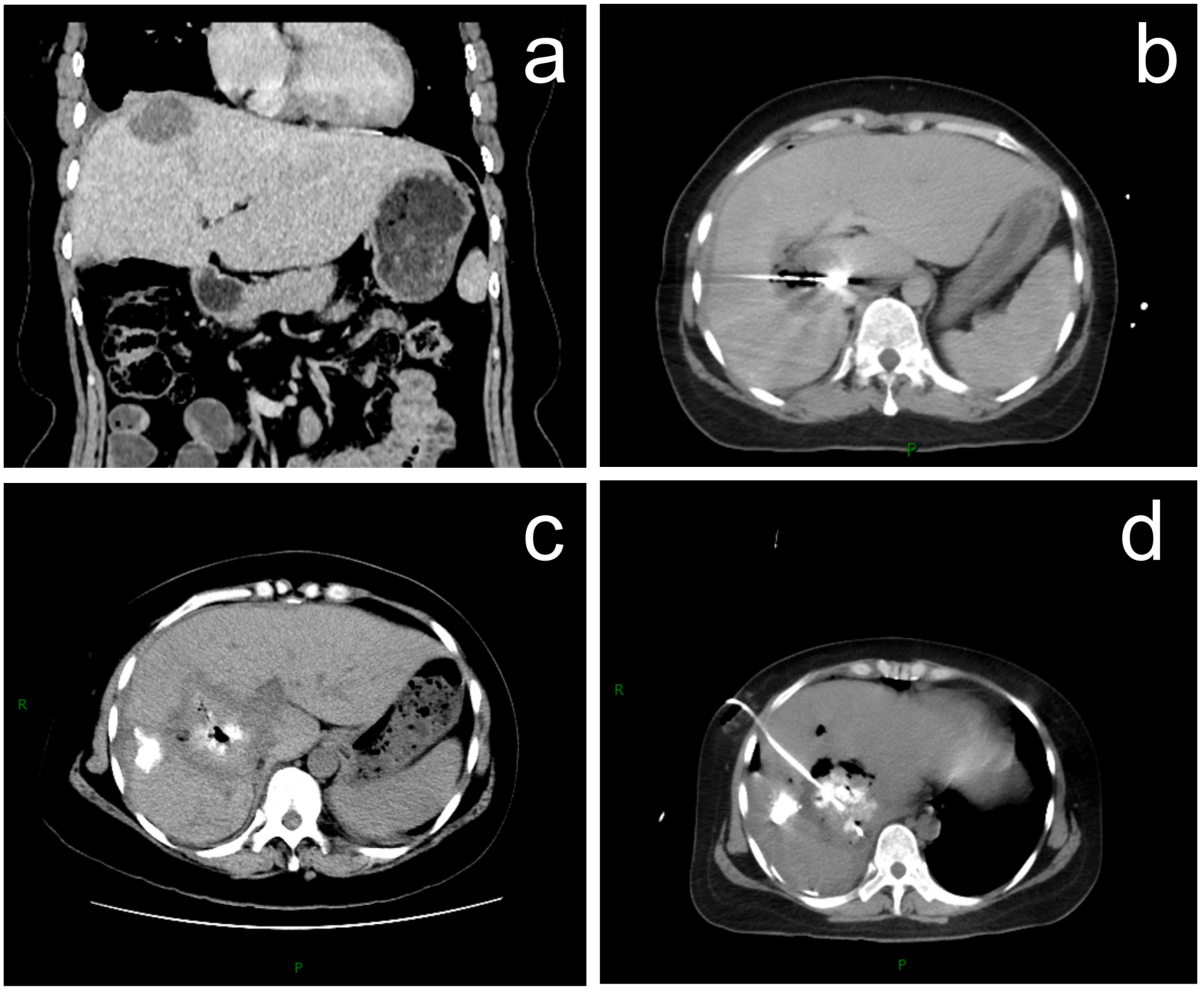
Percutaneous microwave ablation (PMWA) of liver metastases and computed tomography (CT) images of pyogenic liver abscesses (PLA). **(a)** Coronal CT image showing the tumor adjacent to diaphragm (red arrow); **(b)** PMWA procedure for liver metastasis; **(c)** CT image of PLA; **(d)** percutaneous drainage procedure of PLA.

**FIGURE 2:**
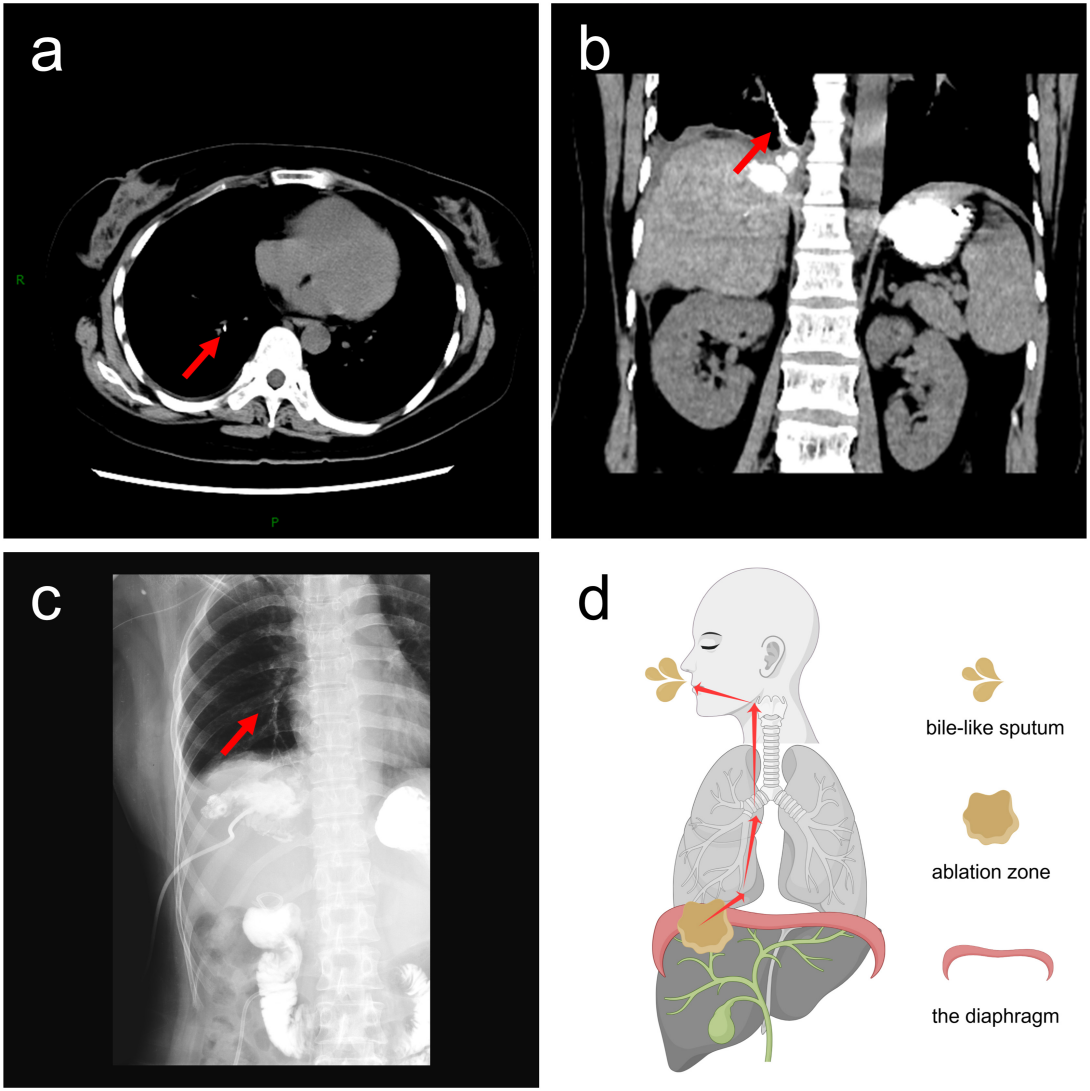
Imaging findings of bronchobiliary fistula (BBF). **(a)** Axial computed tomography (CT) image showing contrast agent within the fistula tract; **(b)** coronal CT image showing contrast agent within the fistula tract; **(c)** plain radiograph showing contrast agent within the fistula tract. The contrast agent within the fistula tract is indicated by red arrows; **(d)** the diagram illustrates the process by which bile passes through the diaphragm into the right segmental bronchus and is ultimately coughed out via the main bronchus and oral cavity. The red arrows indicate the direction of bile flow.

**FIGURE 3:**
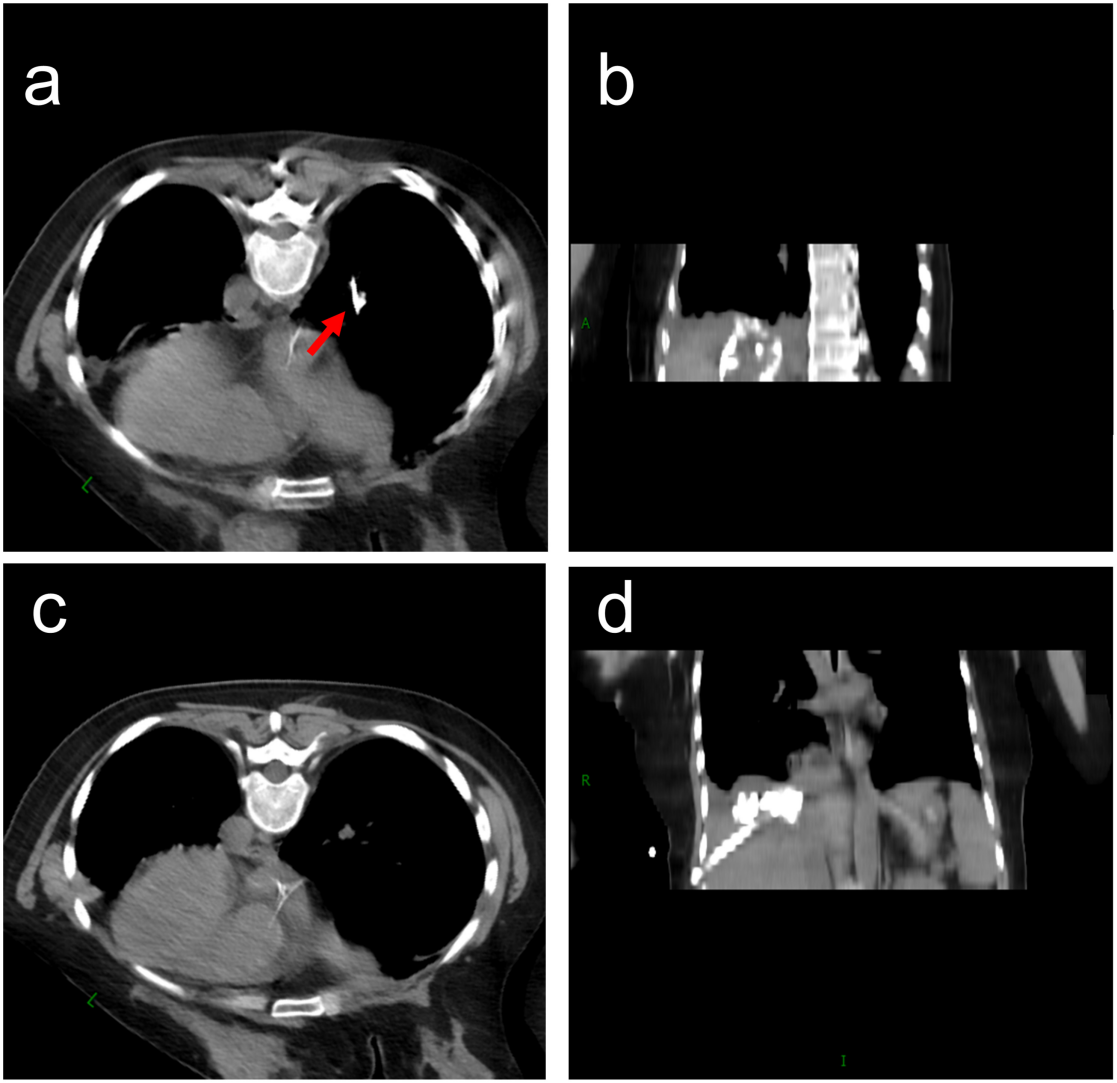
Computed tomography (CT) images of percutaneous microwave ablation (PMWA) for bronchobiliary fistula (BBF). **(a)** PMWA procedure on the fistula tract on October 4, 2022 (red arrow indicates the ablation needle adjacent to the contrast-filled fistula); **(b)** coronal CT image of the fistula after PMWA on October 4, 2022; **(c)** axial CT image of the fistula after PMWA on October 4, 2022; **(d)** repeated coronal CT image of the fistula on October 12, 2022.

A 35-years-old female patient with liver metastases from colon cancer underwent PMWA on August 5, 2022, targeting a lesion in the right hepatic lobe adjacent to the diaphragm (Figures 1a, b). She had no history of chronic obstructive pulmonary disease (COPD), subphrenic bullae, tuberculosis, or bronchiectasis. She developed fever with a maximum temperature of 39 °C on August 6, 2022, and blood cultures revealed *Klebsiella oxytoca*. Contrast-enhanced computed tomography (CT) on August 10, 2022, revealed the formation of a pyogenic liver abscess (PLA) (Figure 1c). Percutaneous drainage of the abscess was performed on August 16, 2022 (Figure 1d). Following antibiotic therapy and drainage, her fever subsided after September 21, 2022. However, she subsequently presented with typical bilioptysis, which was highly suggestive of BBF. To confirm the diagnosis, contrast agent was injected via the drainage tube followed immediately by CT and plain radiography on September 22, 2022. The imaging findings (Figures 2a–d), especially the coronal CT image (Figures 2b, d), clearly demonstrated extravasation of contrast agent from an intrahepatic bile duct branch, forming an abnormal fistula tract through the diaphragm and directly entering the right lower lobe bronchial tree. This imaging evidence was fully consistent with the patient’s clinical symptoms. According to the literature, direct visualization of the fistula tract on cholangiography is a reliable method for the diagnosis of BBF, and detection of bilirubin in sputum can also serve as supporting evidence (9). Therefore, this case was diagnosed as acquired BBF. Despite 13 days of adequate drainage and antibiotic therapy, the patient continued to cough up bile-like sputum. Therefore, on October 4, 2022, PMWA at an ablation watt of 40 w was performed on the fistula tract for 7 min to achieve closure (Figure 3a). A post-ablation CT scan confirmed closure of the fistula tract (Figures 3b, c). A follow-up CT scan on October 12, 2022, again demonstrated closure of the fistula (Figure 3d), and the patient no longer coughed up bile-like sputum.

## Discussion

Bronchobiliary fistula usually results from biliary obstruction, infection, trauma and other pathological processes. Common etiologies include: choledocholithiasis leading to suppurative cholangitis, with subsequent upward spread of infection penetrating the diaphragm; rupture of a bacterial liver abscess through the diaphragm involving the lung and bronchi; and surgical injury to the diaphragm or secondary infection resulting in fistula formation (1). The BBF in the present case is characterized by a clear iatrogenic origin. Its direct cause was thermal injury from the ablation zone and secondary PLA following PMWA of a liver metastasis adjacent to the diaphragm. These factors collectively led to local disruption of diaphragmatic integrity and involvement of adjacent biliary branches, which ultimately led to an abnormal communication between the biliary tract and the bronchial tree. Therefore, this case represents an acquired, iatrogenic fistula, whose pathogenic mechanism distinctly differs from that of classic BBF caused by cholelithiasis or infection.

In this case, according to the biological principles of PMWA, we innovatively applied this treatment for the first time to occlude a BBF. The mechanism for achieving permanent BBF closure lies in a coherent pathophysiological process: first, PMWA targeting the fistula wall induces an instantaneous local temperature exceeding 60 °C through thermal effects, which leads to irreversible protein denaturation and coagulative necrosis of the tissue cells in the fistula wall (10). This is not merely a destructive event, and more importantly, necrotic tissue subsequently triggers the body’s intrinsic repair program, which elicits a strong localized inflammatory response (11, 12). The inflammatory environment promotes the proliferation and migration of fibroblasts toward the lesion site, along with the abundant synthesis and deposition of collagen. Ultimately, the original fistula is completely replaced and obliterated by newly formed, dense fibrous scar tissue, achieving permanent physical closure. This process is similar in principle to that of PMWA for the treatment of solid tumors, but the application goal shifts from the destruction of tumor tissue to sealing an abnormal channel. In terms of this mechanism, PMWA has its unique advantages in sealing a BBF: this technique requires only precise placement of the ablation antenna into the fistula tract to initiate the aforementioned healing cascade with minimal trauma, thereby achieving a minimally invasive, precise, and durable therapeutic effect (13–15).

Of course, the application of PMWA for BBF closure also presents several limitations. First, technical success may be limited for fistulas that are large in diameter or anatomically complex, particularly when they are adjacent to critical vascular or biliary structures, as complete and safe ablation may not be achievable in such cases. Second, to our knowledge, this represents the first reported use of PMWA for this specific condition, therefore, its long-term efficacy and safety profile require validation through larger clinical studies and longer follow-up. In conclusion, this case study demonstrates that PMWA is a novel, minimally invasive, and effective therapeutic alternative for the treatment of BBF. Future work should focus on refining puncture techniques, optimizing energy delivery parameters, and obtaining more robust clinical evidence to further validate the feasibility of this technique.

## Data Availability

The datasets presented in this article are not readily available because of ethical and privacy restrictions. Requests to access the datasets should be directed to the corresponding author/s.
